# An experience of use of canakinumab IL-1 inhibitor in children with cryopyrin-associated periodic syndromes (CAPS)

**DOI:** 10.1186/1546-0096-12-S1-P267

**Published:** 2014-09-17

**Authors:** Svetlana O Salugina, Evgeny Fedorov, Nina Kuzmina

**Affiliations:** 1 Children, Nasonova Research Institute of Rheumatology, Moscow, Russian Federation

## Introduction

Interleukin-1β (IL-1β) is a basic mediator of cryopyrin-associated periodic syndromes (CAPS). In this respect an experience of the use of IL-1 inhibitors has been gained in patients with CAPS. Canakinumab was approved by FDA and EMEA in 2009 and registered in the Russian Federation in 2011 for treatment of CAPS. Canakinumab is shown to have high efficiency and good tolerability in patients with CAPS.

## Objectives

to present an experience of use of canakinumab IL-1 inhibitor in children with CAPS in Russia.

## Methods

The study includes 4 patients with CAPS: two patients with Muckle-Wells syndromes and two patients with CINCA/NOMID syndrome including three girls (3.5, 5.5 and 8 years old) and a boy (17 years old). 1 female patient with MWS received glucocorticoids in a dose of 0.1 mg/kg and other patients received NSAID. All patients passed molecular genetic test to find NLRP3(CIAS1) gene mutation. Two patients with MWS had pThr436Ile and pThr438Ile mutations and two patients with CINCA/NOMID were negative. Canakinumab was administered in a dose of 4 mg/kg for body weight under 15 kg or 2 mg/kg for body weight over 15 kg and injected subcutaneously every 8 weeks. By now, two patients have received 4 injections (32 weeks of observation) and two patients have received 3 injections (24 weeks of observation).

## Results

All patients developed significant clinical improvement: recovery of well-being, relief of fever and rash, reduce in the level of acute phase markers. The effect is stable during the whole follow-up period. This allowed to discontinue glucocorticoid therapy in a female patient with MWS at all. No adverse effects were observed in any patients.

## Conclusion

An experience of the use of canakinumab in patients with CAPS has shown high efficiency and good tolerability of the drug. Decrease in acute phase markers was appeared to be slower in patients with CINCA/NOMID syndrome as the most severe CAPS form.

## Disclosure of interest

None declared.

